# Picene and PTCDI based solution processable ambipolar OFETs

**DOI:** 10.1038/s41598-020-78356-5

**Published:** 2020-12-16

**Authors:** Balu Balambiga, Ramachandran Dheepika, Paneerselvam Devibala, Predhanekar Mohamed Imran, Samuthira Nagarajan

**Affiliations:** 1 Department of Chemistry, Central University of Tamil Nadu, Thiruvarur, 610 005 India; 2 Department of Chemistry, Islamiah College, Vaniyambadi, 635 752 India

**Keywords:** Chemistry, Materials science

## Abstract

Facile and efficient solution-processed bottom gate top contact organic field-effect transistor was fabricated by employing the active layer of picene (donor, **D**) and N,N′-di(dodecyl)-perylene-3,4,9,10-tetracarboxylic diimide (acceptor, **A**). Balanced hole (0.12 cm^2^/Vs) and electron (0.10 cm^2^/Vs) mobility with I_on/off_ of 10^4^ ratio were obtained for 1:1 ratio of **D**/**A** blend. On increasing the ratio of either **D** or **A**, the charge carrier mobility and I_on/off_ ratio improved than that of the pristine molecules. Maximum hole (µ_max,h_) and electron mobilities (µ_max,e_) were achieved up to 0.44 cm^2^/Vs for 3:1 and 0.25 cm^2^/Vs for 1:3, (**D/A**) respectively. This improvement is due to the donor phase function as the trap center for minority holes and decreased trap density of the dielectric layer, and vice versa. High ionization potential (− 5.71 eV) of 3:1 and lower electron affinity of (− 3.09 eV) of 1:3 supports the fine tuning of frontier molecular orbitals in the blend. The additional peak formed for the blends at high negative potential of − 1.3 V in cyclic voltammetry supports the molecular level electronic interactions of **D** and **A**. Thermal studies supported the high thermal stability of **D**/**A** blends and SEM analysis of thin films indicated their efficient molecular packing. Quasi-π–π stacking owing to the large π conjugated plane and the crystallinity of the films are well proved by GIXRD. DFT calculations also supported the electronic distribution of the molecules. The electron density of states (DOS) of pristine **D** and **A** molecules specifies the non-negligible interaction coupling among the molecules. This **D**/**A** pair has unlimited prospective for plentiful electronic applications in non-volatile memory devices, inverters and logic circuits.

## Introduction

Organic electronics is witnessed as a promising young technology with overwhelming advantages such as light-weight, easy processability, flexibility and thus it is a cost-effective alternative to silicon-based semiconductors^1^. Electronics research community is exploring the utility of organic semiconducting molecules in the fabrication of devices including organic field-effect transistors (OFET), organic photo voltaics (OPV), organic light-emitting diodes (OLED), organic photo detectors (OPD) and organic thin film transistors (OTFT)^2–4^. The development of high-performance OFET is in need, since it is a key component of integrated circuits. OFETs are used in applications such as, memory devices, radio frequency identification (RFID) tags, sensors, complementary inverters and smart textiles^5^. However, more practical applications and improvements can be achieved by devoting the research on three key zones of OFET, namely, creating new strategies for high mobility, stability and simplified fabrication procedure^6^. Compared to unipolar devices (*p/n* channel), complementary logics are more efficient and reliable due to less power dissipation and large noise margins^7,8^. In ambipolar devices, the ability to have balanced *n-* and *p-*channel conduction is the basic and important criteria for good performance towards real life applications^9^.

One approach to obtain balanced charge transport with good stability is to integrate two or more components with different electronic properties. Even, exceedingly good outcomes are achieved than the individuals’ electronic properties^10,11^. The integrated electronic property can be attained through either fused D–A system^12–14^ or D–A binary blends^15,16^. Fused D–A systems are renowned as the ubiquitous design, however they involve multistep protocols for synthesis. It is hard to achieve balanced and high mobilities in these fused systems, due to the difficulty in charge carrier injection into the active layer^17^. Blending D and A in appropriate ratio can be a simple and better substitute for fabrication of efficient ambipolar devices. Reports are available on mixing a *p-*channel conjugated polymer with a *n-*channel small molecule to achieve ambipolarity in solution-processed OFETs^18^. Owing to a few disadvantages of polymer, as a substitute, fabrication of ambipolar OFETs employing both *p-* and *n-*channel small molecules has begun^19–21^. One method of generating a thin film with ambipolar characteristics is to make bulk heterojunction (BHJ) films. In this structure, both semiconductors are in contact with dielectric, which forms charge transport channel for both hole and electron at the interface (semiconductor/dielectric layer)^22^. In addition, BHJ semiconductor films can be processed easily from solution in ambient conditions. Solution processability renders mono-dispersity and well-defined microstructures of small molecules in low cost^23^. There have been a few reports on solution processable ambipolar OFETs using small molecular BHJ architecture^24,25^. While designing an ambipolar transistor via BHJ, keen attention must be given to the highest occupied molecular orbital (HOMO) and lowest unoccupied molecular orbital (LUMO) levels of the chosen materials^26^.

**Figure 1:**
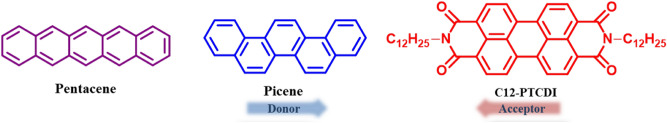
Molecular structures of pentacene, picene (**D**) and C12-PTCDI (**A**).

In this regard, we report high performance BHJ ambipolar OFET device using picene as donor and N,N′-di(dodecyl)-perylene3,4,9,10-tetracarboxylicdiimide (C12-PTCDI) as acceptor (Fig. 1). Picene is a phenacene and the structural isomer of pentacene^27^. Pentacene is very well-known benchmark *p*-channel molecule and regarded as a small unit of graphene. This isomers display contrasting optical and electronic properties due to different ring arrangements that changes the energy levels of the conjugation. Pentacene possess *zig-zag* arrangement of the benzene rings (symmetry: D_2h_) and picene has armchair edges (symmetry:C_2v_)^28^. According to Clar’s aromatic sextet rule armchair edge has more aromatic stability than *zig-zag* edge. This fact accounts for the high thermodynamic stability of picene^29^. It has greater intrinsic stability than pentacene under atmospheric conditions owing to its deep HOMO level (− 5.5 eV) and optimum band gap (*E*_g_ = 3.3 eV). The hole mobility of picene is up to 1.1 cm^2^/Vs^30^. Interestingly, field-effect characterization of picene based devices showed the increase in I_on/off_ and mobility after exposed to air (O_2_) which specifies the combination of oxygen had improved the transport^31^. While pentacene is sensitive to air and light, especially in the internal ring due to its narrow bandgap and high HOMO energy level^32^. Another important advantage of picene is its better solubility than pentacene in common organic solvents. In spite of these favorable features, limited work has been reported on picene based organic electronics. PTCDI belongs to a class of perylene derivatives, which are well-studied electron-transporting material, forms *n-*channel semiconductors with high electron affinity, good charge carrier mobility, solubility, excellent thermal and photostability^33^. It is chosen as the *n-*channel counterpart for the heterojunction.

A series of blend ratio of picene/C12-PTCDI were investigated for their interaction in terms of photophysical, electrochemical and OFET behaviors. Through tuning the ratio of the **D/A** blends, stable and efficient intermolecular stacking modes were obtained. The unique behavior of the blend and films were studied systematically using scanning electron microscope (SEM), optical spectroscopic techniques, thermal studies and grazing incidence X-ray diffraction (GIXRD). We anticipate the different crystalline microstructures and morphologies with respect to **D/A** blends will influence the OFET behavior. For highly balanced and efficient ambipolar charge transport, direct injection of charge carriers to active layer from the electrodes is desirable. Since, both the *p-* and *n-*channel semiconductors are in direct contact with source and drain electrodes in BHJ system. These blends can significantly exert different ambipolar OFET behavior with improved charge carrier mobilities.

## Results and discussion

### Synthesis of picene and C12-PTCDI

**Scheme 1:**
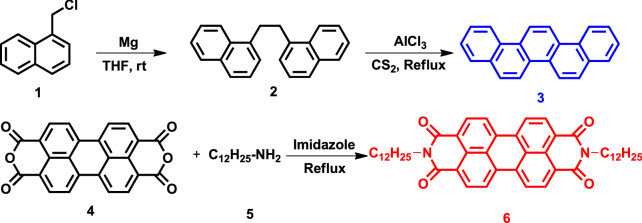
Synthesis of picene and C12-PTCDI molecules.

The synthetic routes of picene and C12-PTCDI are presented in Scheme 1. The detailed synthetic procedures of precursors and final molecules are given in the supporting information (SI). Briefly, picene (**3**) was synthesized via insitu Grignard reaction of 1-chloromethylnaphthalene (**1**), followed by cyclodehydrogenation of dinapthylethane (**2**). Through the direct imidazation reaction between the perylene dianhydride (**4**) and dodecylamine (**5**), gave symmetric bisimide N,N′-di(dodecyl)-perylene 3,4,9,10-tetracarboxylic diimide (**6**) in high yield. The NMR and HRMS spectra of **3** and **6** are given in Figures S12−S17 (supporting information).

### Photophysical properties

**Figure 2:**
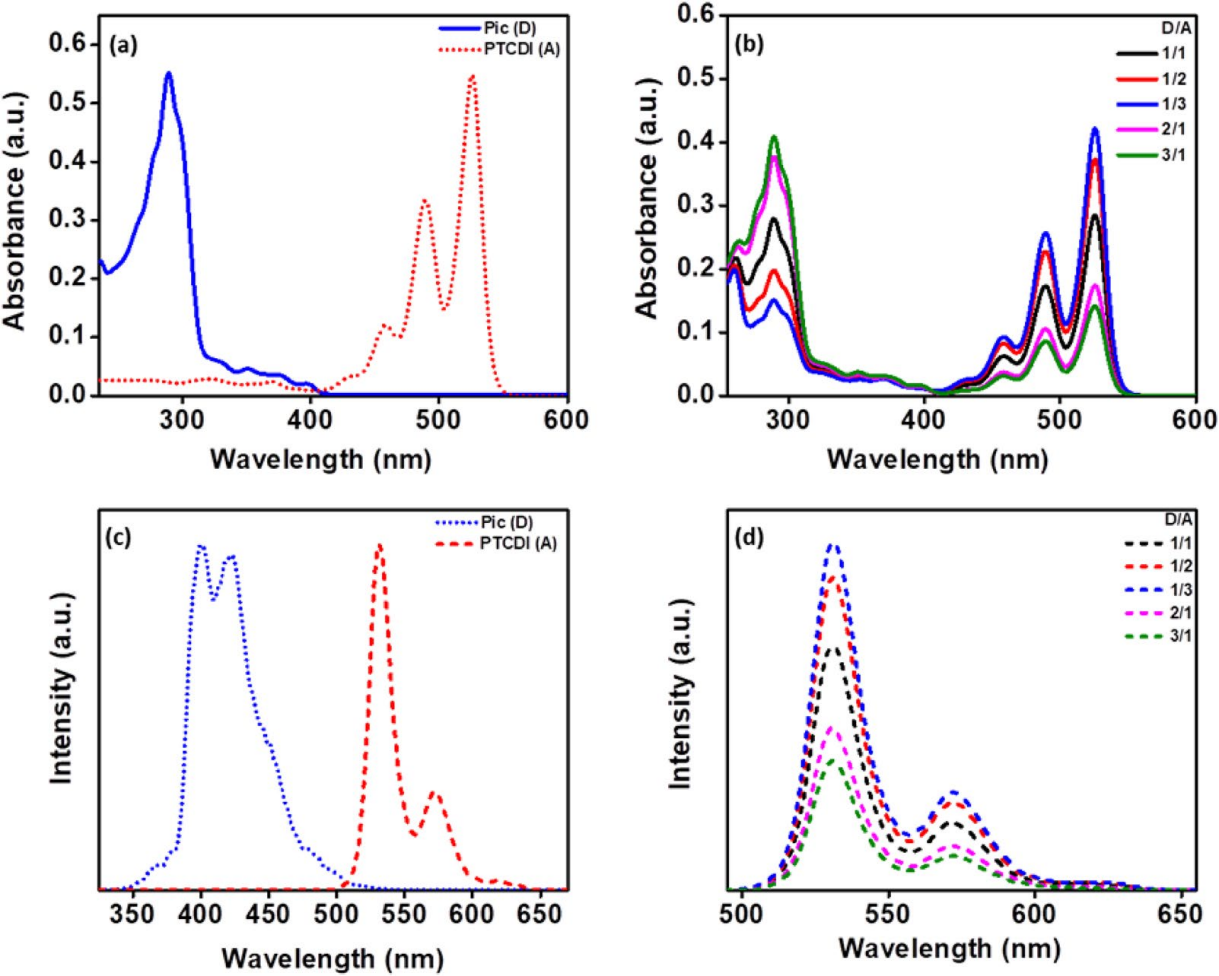
Absorption (**a**, **b**) and emission (**c**, **d**) spectra of **D, A** and **D/A** blends. Graphs were plotted using Origin software (https://www.originlab.com/, version: 8.5).

UV–Vis absorption and emission spectra of the compounds **3** (**D**), **6** (**A**) and **D**/**A** blends were investigated to understand the photophysical properties. UV–Vis spectra were recorded at a low (10^−5^ M) and high concentrations (10^−3^ M). From the absorption spectra three distinct peaks (450–525 nm) were observed for **A**, corresponds to 0–2, 0–1 and 0–0 transitions. In addition a shoulder peak near 425 nm was found and accounted for 0–3 electronic transition of C12-PTCDI^34^. Picene showed maximum absorbance wavelength of 289 nm, attributed to 0–0 transition (Fig. 2a)^35^. With the increase of **D** proportion the intensity of higher energy transitions enhanced (Fig. 2b). However, on comparison with pristine **D** and **A** in CHCl_3_, the **D**/**A** blends have not shown any noticeable shift in absorption. The emission behavior of the individual and blends was measured through PL-spectroscopy, at 10^−7^ M and 10^−5^ M concentrations. Figure 2 depicts the emission spectra of **A** where the peak shape resembles the absorption spectral pattern (mirror image)^36^. The emission behavior of blend systems showed the pattern similar to that of pristine **A**, and there is no shift or new peak observed in the spectra (Fig. 2d). Peak position shifts were not even found in concentrated blends of **D** and **A** (Figure S1, supporting information). From the absorption and emission studies visible charge transfer between picene and C12-PTCDI are not observed^37^.

### Electrochemical properties

**Figure 3:**
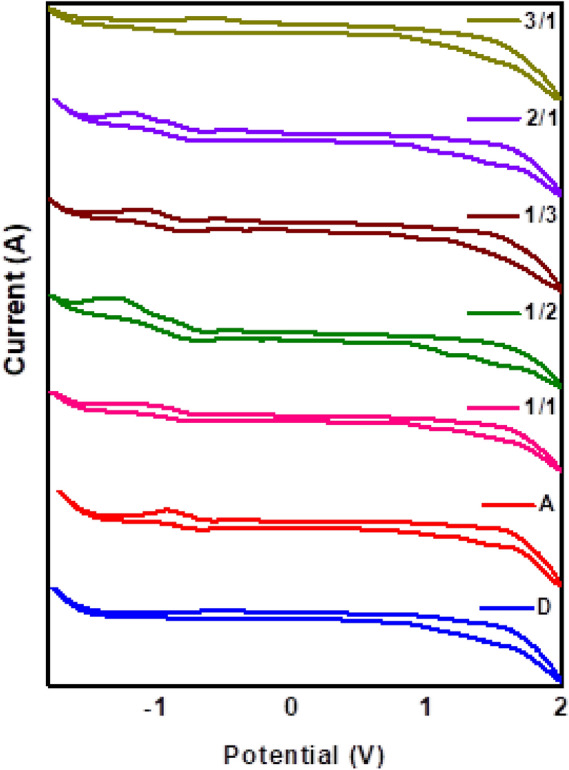
Cyclic voltammograms of pristine **D**, **A** and **D/A** blends Graph was plotted using Origin software (https://www.originlab.com/, version: 8.5).

To understand more about the molecular level electronic interactions between **D** and **A**, electrochemical properties were explored using cyclic voltammetry (CV). All the experiments were performed at 100 mV/s scan rate with 0.1 M tetrabutylammonium hexafluorophosphate (Bu_4_NPF_6_) as supporting electrolyte in chloroform. Ferrocene served as the external standard. As shown in Fig. 3, **D** and **A** illustrated significant curves with the oxidation and reduction onsets, and the corresponding ionization potential (IP)/electron affinity (EA) values were calculated^38^ and tabulated (Table 1). The CV curves remain unchanged even after multiple scans. The CV of **D** and **A** showed the first oxidation potential at 1.41 and 1.70 V, and the corresponding IP/EA values were calculated to be − 5.81/− 2.57 and − 6.10/− 3.58 eV, respectively. Thus, the fundamental gap of the pristine **D** or **A** was lower than the intermolecular gap between the **D** and **A.** Consequently, the redox behavior of the **D/A** blends for different proportions was also examined. Different proportions of **D**/**A** blends exhibited comparable oxidation and reduction peaks with different peak potentials^39^. Remarkably, an additional peak was observed at higher negative potential for the blends at around—1.3 V, which ensures the intermolecular electronic interaction^40^. IP and EA values of blends allowed an effective comparison of the modification of energy levels among the various **D**/**A** proportions. The 3:1 ratio, observed with higher value of IP and lower band gap than other D/A blend ratios, which ensure the better hole injection. Likewise, for 1:3 ratio shows low lying EA value of − 3.09 eV. On increasing the proportion of **A** in the blends respective IP values reduced, it increases the energy barrier for hole injection and thus *p-*channel transport efficiency will reduce considerably^41^.

**Table 1:** Electrochemical data of **D**, **A** and **D/A** blends.

D/A ratio	E_ox_(V)	E_red_(V)	Experimental (eV)		
			IP	EA	IP-EA
Picene	1.41	− 1.83	− 5.81	− 2.57	3.24
C12-PTCDI	1.70	− 0.82	− 6.10	− 3.58	2.52
1:1	1.38	− 1.35	− 5.78	− 3.05	2.73
1:2	1.39	− 1.33	− 5.79	− 3.07	2.72
1:3	1.39	− 1.31	− 5.79	− 3.09	2.70
2:1	1.33	− 1.35	− 5.73	− 3.05	2.68
3:1	1.31	− 1.34	− 5.71	− 3.06	2.65

### Thermal behavior

**Figure 4:**
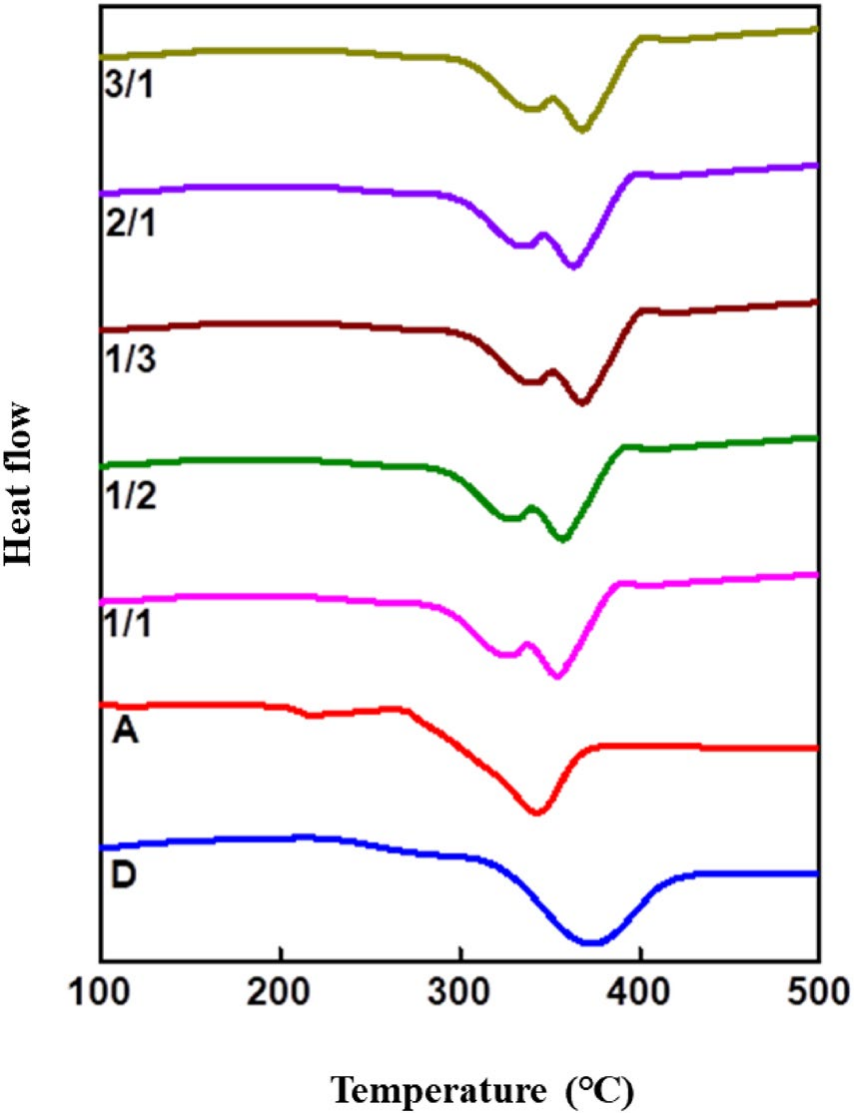
DSC thermograms of pristine **D**, **A** and **D/A** blends. Graph was plotted using Origin software (https://www.originlab.com/, version: 8.5).

Differential scanning calorimeter (DSC) and thermogravimetric analysis (TGA) were employed to study the robustness and thermal stability of the semiconducting materials. The experiments were performed at a heating rate of 10 °C/min in N_2_ atmosphere. **D** and **A** exhibited melting points (T_m_) of 367 and 340 °C, correspondingly, representing a good thermal endurance ability of the molecules, this assure a stable **D**/**A** blend film for OFET device (Fig. 4**)**. Thermal decomposition temperatures (T_d_, with 10% weight loss) of **D** and **A** were estimated to be 386 and 441 °C, correspondingly, further informs the good thermal properties of these systems for a preferred OFET application (Figure S7, supporting information).

To understand the thermal behavior of **D**/**A** blended systems, five different ratio of **D**/**A** were taken by weight. The dependence of the characteristics of DSC and TGA curves on the blend composition is shown in Table 2. DSC thermograms and TGA curves of **D/A** blends shows different melting and decomposition temperatures for **D**/**A** blends than the pristine molecules. Hence **D** and **A** are miscible at molten state the DSC analysis proves the coexistence of crystalline phases of both materials in the blend. This can explain formation of ambipolar channels for holes in **D** and electrons for **A** in blended OFET devices^42^. Moreover the decomposition temperature of donor also varied with the change in acceptor ratio in the blends. The decomposition of all **D**/**A** ratio is found to be increased than the pristine **D** with respect to the increase in the acceptor fraction. The thermal stability of **D** and **A** in the blend is significantly changed due to possible interaction of the **D**/**A** blend^43^. This will alter and improve the thermal properties while blending them for device applications.

**Table 2:** Thermal properties of **D, A** and **D/A** blends.

D/A ratio	T_m_ (°C)	T_d_ (°C)
**D**	367	386
**A**	340	441
1/1	326,352	390
1/2	359,328	392
1/3	360,338	395
2/1	363,334	387
3/1	364,333	389

### Thin film XRD analysis

**Figure 5:**
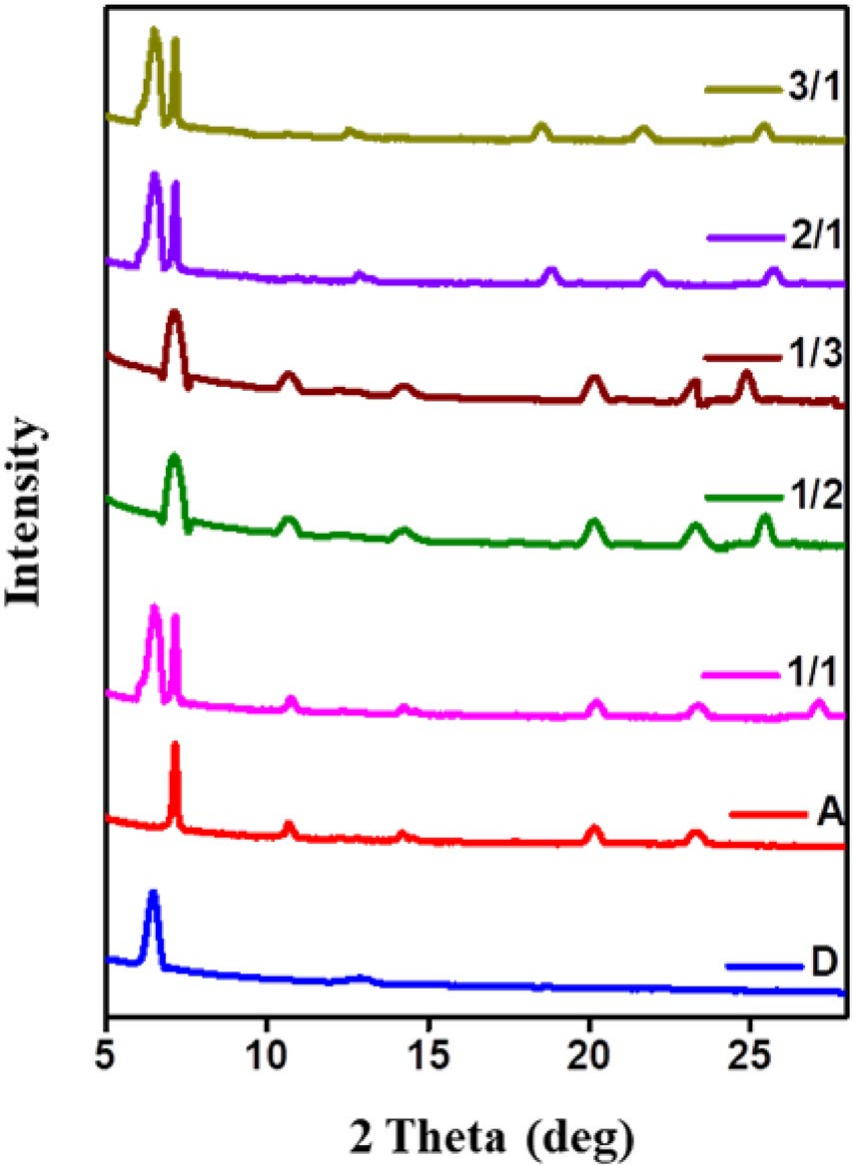
Thin film XRD patterns of **D,A** and **D/A** blends, Graph was plotted using Origin software (https://www.originlab.com/, version: 8.5).

In order to examine the structural and crystalline orders of the pristine and blend films, GIXRD analysis was performed on thin films annealed at 90 °C and the molecular stacking patterns are represented in Fig. 5. The thin film of pristine **D** showed a peak at 6.50° and 12.87°and the corresponding d-spacing value found to be 13.58 Å and 6.87 Å respectively^44^. While for pristine **A**, well-defined XRD peaks were witnessed at 7.10°, 10.69° and 14.28°, with the d-space of 12.42 Å, 8.26 Å and 6.19 Å. The 20.13° peak is indexed to the edge-to-edge distance between the two stacked **A** planes**,** which make a tilted packing conformation. The d-spacing of 3.81 Å (obtained from peak 23.33°) corresponds to the typical π–π stacking distance as common for planar conjugated systems^45^.

To gain more insight on the molecular arrangement of blend films, XRD peaks were detailed in the same method. For the 1/1 film of **D**/**A**, the XRD pattern shows seven diffraction peaks at 2ϴ, 6.47°, 7.18°, 10.69°, 14.28°, 20.29°, 23.41° and 27.08°, illustrating an effective interaction between picene and C12-PTCDI to produce a preferred co-crystal stacking^46^. A new peak is observed in all the five **D/A** blends and the d-spacing of the peak is slightly changing with **D** to **A** ratio. The d-spacing ranges from 3.30 to 3.40 Å could be recognized as the quasi-π-π stacking owing to the large π conjugated plane of **D** and **A**. Though, with the increasing C12-PTCDI portion (1/2 and 1/3), the XRD analysis exhibited peaks consistent with the thin film of pristine **A**. Thus signifies a desired long-range ordering of the **A** in blends, that is mostly brought by the more amounts of branched alkyl chains with a large steric hindrance that prevented the ordered stacking of **D**^47^. Overall the crystallinity is reserved in BHJ blended films. The observation of peak broadening, along with a decrease in d-space value proposes that **D** is interrupting the **A** network and thus forming the desired hole and electron transporting domains for ambipolar OFET devices^48^.

### Morphological analysis of thin films

**Figure 6:**
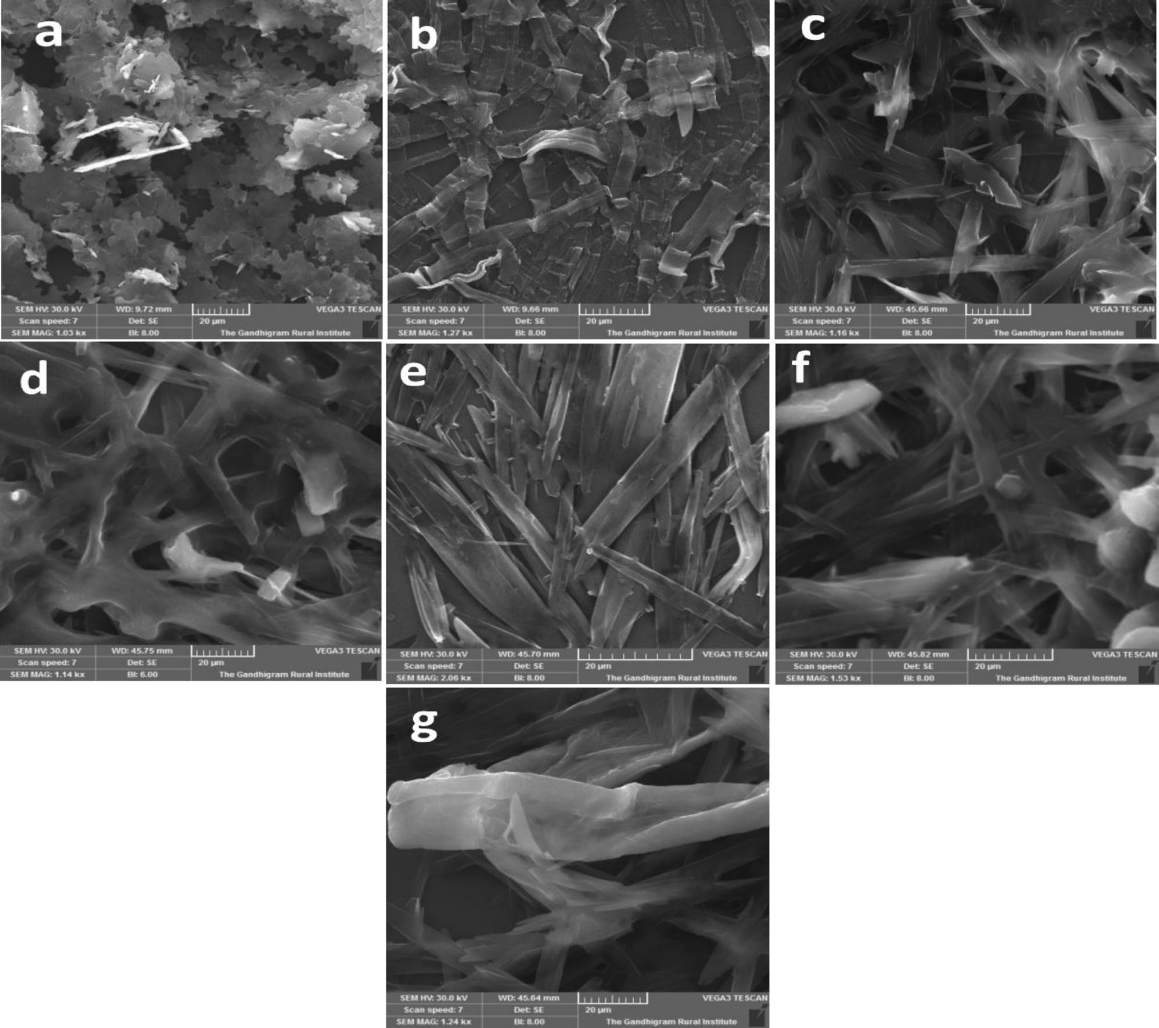
SEM images of **D, A** and **D/A** blend films (**a**) **D** (**b**) **A** (**c**) 1/1 (**d**) 1/2 (**e**) 1/3 (**f**) 2/1 (**g**) 3/1 with scaling of 20 µM.

The microscopic structure of the film has the obvious influence on the transistor performance. To explore this dependency, SEM investigations were performed on thin films of pristine and **D**/**A** blends. Films are spin coated on cleaned Si-wafers with chloroform at 3200 rpm for 50 s and heated to 90 °C for the removal of remaining solvent. The active layer topography of the devices with **D**, **A** and five **D**/**A** blends were analyzed by SEM. From the Fig. 6**,** it is observed that all films are highly crystalline. Picene is composed of layer-by-layer flakes morphology with complete area coverage. This crystalline flake-like nature appeared as a result of intermolecular *π*-*π* interaction between aromatic core of picene. C12-PTCDI shows nanobelt like structure that grow in all directions with uniform nature. The 1D molecular assembly of **A** is accounted for the hydrophobic nature of the long alkyl chains greatly helpful for the π-π interaction between perylene planes^49^. The self-assembly patterns observed in the blend films are completely different from the pristine films^50^.

Blends’ morphologies are all uniform and well-connected. The rod shaped morphology is obtained from 1/1 and 1/2 blends of **D** and **A**. The ribbon shaped micro structure is exhibited by 1/3 ratio and fiber morphology is shown by 3/1 blend of **D**/**A**. These microstructures were intertwined in nature. All these specified morphologies with high coverage and good topographical arrangement in thin film intensely certifies unbroken packing and eventually warrants the improved charge injection and transport^51^.

### Proposed molecular stacking

**Figure 7:**
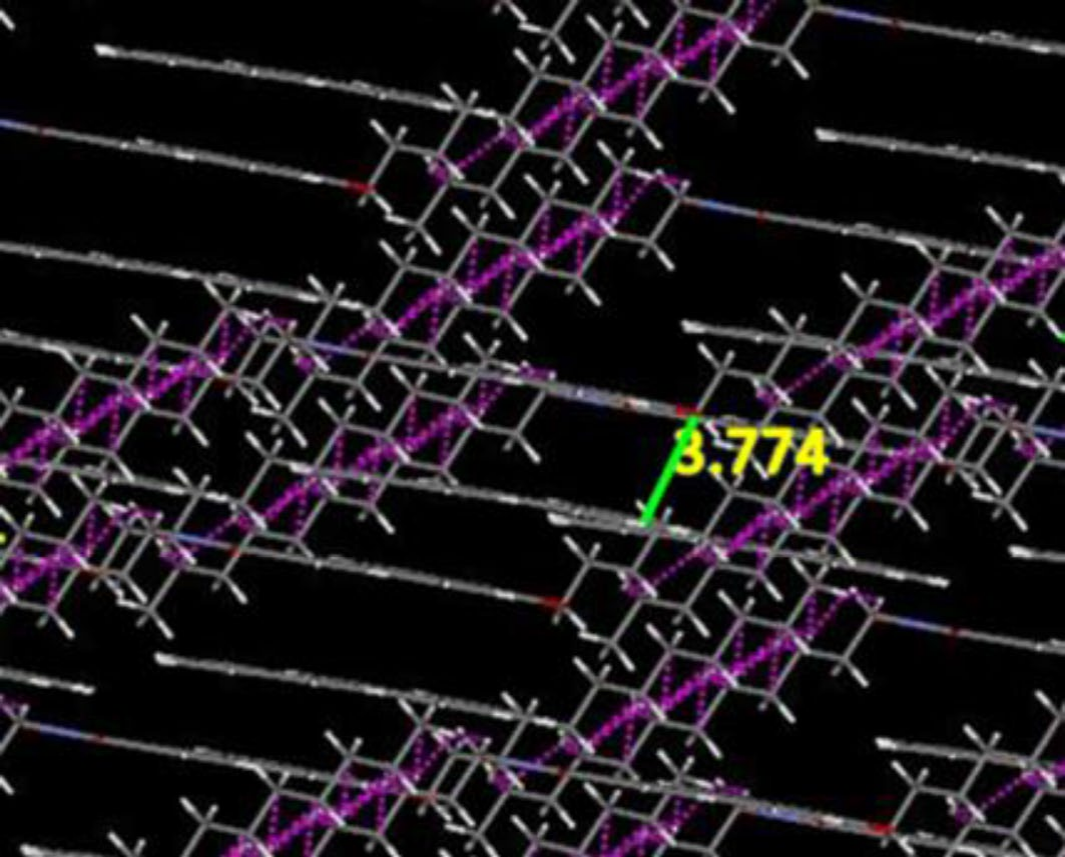
The stacking patterns of 1:1 **D/A** blends at MedeA VASP version 1.0 using VASP 5.3.3 (https://www.vasp.at/).

As per the molecular mechanics calculation of the donor–acceptor molecule using MedeA's VASP, the packing of the D–A molecule was done with the PBC of a = 40, b = 17 and c = 15 over triclinic pattern. Very compact packing was obtained for D/A in 1:1 ratio. The pink lines represent available hopping distance for H-atoms. The other measured H-bonding distances in the intermolecular pattern confirm the stability of the D–A packing. The distance between D and A is around 3.774 Å as shown in Fig. 7.

### OFET characterization

**Figure 8:**
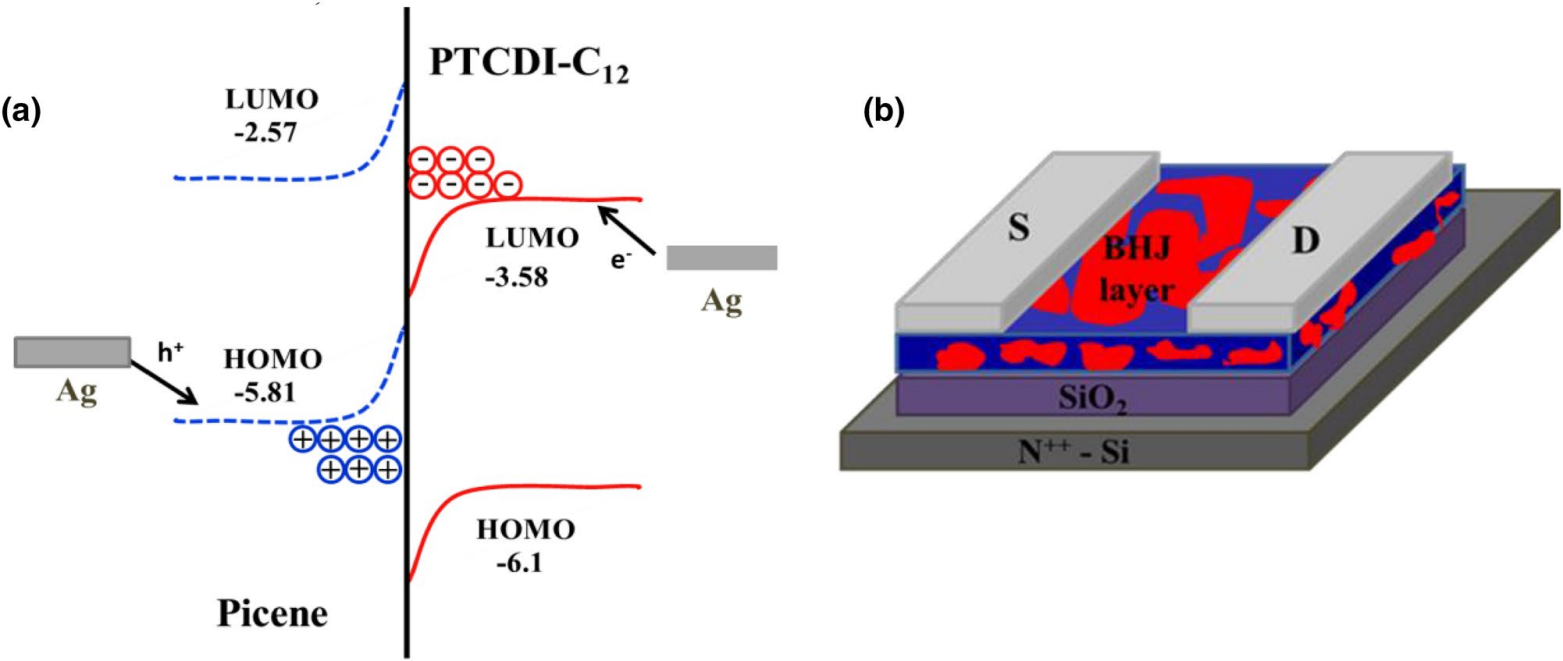
(**a**) Energy level diagram for picene—C12-PTCDI system (**b**) schematic illustration of the BHJ OFET device. Figures were drawn in Microsoft Office PowerPoint (https://www.microsoft.com/en-in/microsoft-365/powerpoint).

The charge transporting ability of pristine donor, acceptor and the heterojunction **D**/**A** blend films is scrutinized via OFET device performance. OFET devices were fabricated in BGTC architecture (Fig. 8b) by spin coating from chloroform solution. The top contact ensures the uniform orientation of the active layer over dielectric layer where in the bottom contact devices, the orientation over electrodes and dielectric layer varies and thus disturbs the charge carrier movement. Therefore, minimum contact resistance is obtained in BGTC architecture^52^. The fabricated devices were characterized in ambient conditions; threshold voltage (V_TH_) and charge carrier mobility (µ) were obtained from the intercept and slope of the linear plot of the square root of drain to source current (I_DS_^1/2^) versus the gate voltage. The below formula was applied to calculate charge carrier mobility extracted from the saturation regime^53^.

$$\mu = \frac{2L}{{CW}}\left( {Slope} \right)^{2}$$

where L and W are length and width of the channel, C is capacitance of the dielectric layer per unit area, correspondingly. In organic hetero structured devices, energy level alignment is an influencing factor to study the carrier processes such as transport, recombination, and separation at the interface. BHJ devices are commonly systematized in three ways: straddling gap (type I), staggered gap (type II) and broken gap (type III). Here, staggered energy level alignment was used and the structure is schematically represented in the Fig. 8a. A unique feature of the staggered device is, the energy levels of **D** and **A** are stacked with a partial overlap^54^.

**Figure 9:**
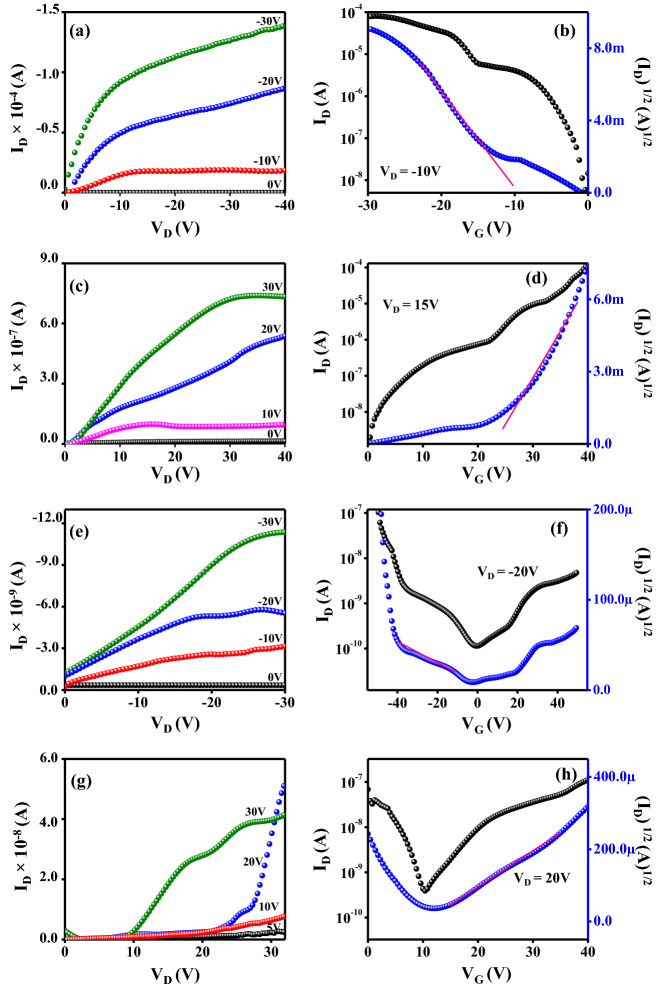
OFET characteristic curves of **D** (**a**, **b**); **A** (**c**, **d**); *p-*channel (**e**, **f**) and n-channel (**g**, **h**) of 1/1 blend. Graphs were plotted using Origin software (https://www.originlab.com/, version: 8.5).

The measured hole mobility for the pristine picene (**D**) was 0.38cm^2^/Vs with good I_on/off_ ratio of 10^5^, which is on par with the reports using various processing techniques^55^. The electron mobility of pristine C12-PTCDI (**A**) was up to 0.12 cm^2^/Vs with I_on/off_ ratio of 10^4^ (Fig. 9)^56^. Further, the bulk-heterojunction OFETs were fabricated from the blended solution of **D** and **A** in CHCl_3_. The blend solution preparation involves mixing the two semiconductors in the ratio of 1/1, 1/2, 1/3, 2/1 and 3/1 and devices were fabricated following the same procedure mentioned for the pristine devices. The linear and saturation regime of the output curves are differently observed for 1/1 ratio than the unipolar devices. This observed superposition of standard saturated behavior at high V_g_ and a superlinear current increase at low V_g_ and high V_ds_ is due to the injection of opposite carriers^57^. The resulting OFETs were characterized and the parameters are given in Table 3. The representative output and transfer curves are shown in Fig. 9 and Figures S8–S11 (supporting information).

**Table 3:** OFET characteristics of **D, A** and **D/A** blends*.

S. no	*p*-channel^a^			*n*-channel^a^		
	μ_h_ (cm^2^/Vs)	I_on/off_	V_Th_ (V)	μ_e_ (cm^2^/Vs)	I_on/off_	V_Th_ (V)
Picene	0.38	10^5^	− 9	–	–	–
C12-PTCDI	–	–	–	0.12	10^4^	10
1D/1A	0.12	10^4^	− 13	0.10	10^3^	11
1D/2A	0.13	10^3^	− 7	0.17	10^4^	13
1D/3A	0.12	10^3^	− 10	0.25	10^6^	9
2D/1A	0.41	10^4^	− 11	0.10	10^3^	6
3D/1A	0.44	10^6^	− 12	0.06	10^2^	8

^a^Mean values of five devices.

In the blend film devices, both hole and electron transfer (ambipolar behavior) was observed as expected. Holes accumulate at the semiconductor/SiO_2_ interface when negative gate bias is applied, and the device works as a *p*-channel OFET. While for the positive gate bias, electrons gather at the interface and the device functions as an n-channel OFET. The movement of the carriers and transport pathways of the heterojunction films greatly depends on the ratio of **D** and **A** in the blend; which is due to the crystallinity or transport pathways of the heterojunction films. Among the ratio analyzed, 1/1 blend shows balanced charge carrier transport of 0.12 and 0.10 cm^2^/Vs for hole and electron mobilities, respectively. These values are less than the pristine **D** and **A** devices and this behavior is accounted for the interpenetrating networks of *p* and *n*-channel materials used. The percolating network will conduct either one of the carrier (hole or electron) predominantly due to the varying electron affinities and ionization potentials. Consequently, the resulted charge carrier mobilities of both holes and electrons are lower than the neat films^58^.

**Figure 10:**
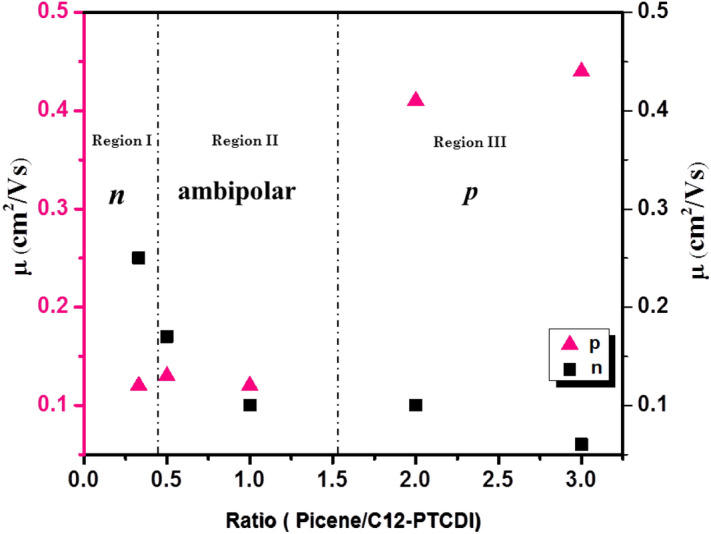
Varying mobilities plotted against **D** to **A** ratio for BHJ ambipolar OFET devices. Graphs were plotted using Origin software (https://www.originlab.com/, version: 8.5) and Microsoft Office PowerPoint (https://www.microsoft.com/en-in/microsoft-365/powerpoint).

In order to show the charge transporting ability of the devices with different **D** to **A** ratio, a plot is represented in Fig. 10**.** Depending on the **D**/**A** ratio, the devices display three operating modes: In region **I**, *n*-channel behavior is more, region **II** is occupied by both n and p channel (ambipolar) transport and in region **III,** *p*-channel behavior is prevailing. Both the pristine **D** and **A** has shown clean *p* and *n* channel behaviors, respectively. When the ratio of **D** to **A** was less than 0.33 (1/3), the device exhibits higher order magnitude of *n* channel behavior than *p-*channel (region **I**). This may be due to the abundance of acceptor increasing the n-channel pathway^59^. Further increasing the **D** to **A** ratio 0.5, (region **II**), *p*-channel behavior is overriding throughout the channel. In this region, ambipolar transport is observed for the ratio of 0.5 (2/1) and 1 (1/1) with significantly improved hole mobility than their pristine films. In these cases, with increase of **D** or **A** fraction, hole or electron mobility is expected to improve. In 1/2 and 1/3 blends, the acceptor fraction is high, the electron mobility is improved from 0.12 (pristine **A**) to 0.17 and 0.25 respectively. This can be understood on the phenomenon of *p* doping for the enhancement of *n* channel OFET characteristics. The *p-*channel material served as a trap for marginal number of holes and helped to decrease the trap density, OFET performance is enhanced^60^. Accordingly, in 2/1 and 3/1 blends (Region **III**), where the donor fraction is two to three times higher than the acceptor showed high hole mobility. The 2/1 ratio of **D/A** showed the hole and electron mobility of 0.41 and 0.10 cm^2^/Vs, respectively. Likewise, 3/1 blend shows the highest hole mobility of 0.44 cm^2^/Vs which is higher than pristine picene.

Moreover, when the ratio is varied from 1/1 (1/2, 2/1, 1/3 and 3/1) slightly unbalanced mobilities are observed. This behavior is explained by the contact of BHJ active layer with electrode: when the **A** fraction is high, electron mobility is pronounced, because the contact area between **A** and electrode is relatively higher than **D** and the vice versa^61^. Further, the **D**/**A** ratio is varied to 1/5 and 5/1 and no ambipolar behavior is observed. Here, one component (**D** or **A**) is five times higher than the other, hence one type of charge carrier (hole or electron) is exclusively transported and the device acted as unipolar^41^. This observation reveals that, optimum ratio between **D** and **A** is highly anticipated to arrive at balanced ambipolar behavior. The charge carrier trapping at the interface (**D** and **A**) have disturbed the threshold voltage values^62^.

## Conclusion

The fabrication of BHJ ambipolar transistors with picene (**D**) and C12-PTCDI (**A**) through solution processing is demonstrated. High ionization potential (− 5.71 eV) of 3:1 and lower electron affinity of (− 3.09 eV) of 1:3 supported the fine tuning of frontier molecular orbitals in the blends. Five blending ratio between **D** and **A** have formed phase-separated networks and three operational regimes. Development of efficient and highly stable ambipolar OFETs was achieved in air without encapsulation. Significant mobility improvement of individuals was obtained. OFETs with mobility up to 0.44 cm^2^ V^−1^ s^−1^ for hole and 0.25 cm^2^ V^−1^ s^-1^ for electron is observed. At an optimized 1/1 ratio, the device displayed an excellent organized network along with balanced hole and electron mobilities. These devices can be potentially employed as active layers in non-volatile memory devices, sensors and inverters with a large transfer gain.

## Experimental section

### Materials

1-Chloromethylnaphthalene, aluminium chloride, perylene dianhydride, imidazole and dodecylamine were procured from Alfa Aesar and Sigma Aldrich. Analytical grade solvents were used as acquired from the commercial sources.

### Methods

^1^H Nuclear magnetic resonance spectra (NMR) were recorded in a Bruker 400 MHz spectrometer in CDCl_3_. UV–Vis absorption spectra were measured using a JASCO UV-NIR spectrophotometer. Fluorescence spectra of the compounds were attained using the Perkin Elmer Fluorescence spectrometer LS-55. Electrochemical characterization was performed using CH 6035D work station. A conventional cell setup containing three electrodes was used with glassy carbon (working electrode), standard calomel electrode (reference) and a platinum wire as the counter electrode. The system was standardized externally using Fc/Fc^+^. High-resolution mass spectra (HR-MS) were recorded in ThermoExactivePlus UHPLC-MS. Thermal analyses were performed under nitrogen flow in Perkin Elmer STA system. SEM measurements were done with a VEGA 3 TESCAN microscopy. Grazing incidence X-ray diffraction was performed in the reflection mode (CuK_α_ radiation) by a XPERT-PRO X-Ray diffractometer. DFT studies were employed to analyze the geometry and energy levels of the molecules. All the details are given in supporting information (Figures S2–S6 and Tables S1–S3).

### OFET device fabrication and characterization

OFETs based on bulk heterojunction blends of picene and C12-PTCDI was fabricated using heavily n^++^ doped silicon wafer in bottom gate top contact (BGTC) architecture. Initially, silicon wafers were ultrasonically cleaned in acetone, methanol and finally over a mild piranha solution. Subsequently, the silicon wafer was heated up to 1200 °C for 80 min to grow SiO_2_ dielectric layer. Thickness of the thermally grown dielectric layer was ~ 300 nm and silicon wafer functioned as gate. The pristine **D**, **A** and different proportions of **D/A** blends were well-dissolved in chloroform and sonicated for 20 min with 5 mg/ml concentration. This solution was layered over SiO_2_ dielectric layer by spin coating at 3000 rpm speed for one minute. Then the device was heated over a hot plate at 60 °C for 45 min to remove residual solvent. In addition, the wafer is thermally annealed at 90 °C for 30 min as a post deposition treatment to attain better self-assembly. Then silver was used to make source and drain contacts to complete the fabrication. The width and length of the channel were 5 mm and 150 µm, respectively.

Keithley 4200 SCS analyzer was used to investigate transistor characteristics. Probe station was utilized to source a voltage and to read back the associated current simultaneously. The SMUs were in turn connected to a probe station (Everbeing), which consist of three test probes, three triaxial wires and micropositioners (made up of tungsten). A test probe is used in each one of terminals (source, drain and gate) of the transistor. Test probes could be adjusted in various directions with the micropositioners and allowed the measurements to characterize the OFETs.

## Supplementary information

Supplementary Information.

## Data Availability

The detailed synthetic procedures, high concentration UV spectra, computational studies, TGA analysis, transistor characteristics (1/2, 1/3, 2/1, 3/1), NMR and HRMS and spectra of compounds are given in supporting information.
