# Supplementary material for: Picene and PTCDI based solution processable ambipolar OFETs
Source: Sci Rep. 2020 Dec 16;10:22029. doi: 10.1038/s41598-020-78356-5 (PMC7744517; doi:10.1038/s41598-020-78356-5)
Supplement: Supplementary file 1 — Supplementary Information. [file 41598_2020_78356_MOESM1_ESM.docx]

Electronic Supplementary material

for

**Picene and PTCDI based solution processable ambipolar OFETs**

**Balu Balambiga**^1^**, Ramachandran Dheepika**^1^**, Paneerselvam Devibala**^1^**, Predhanekar Mohamed Imran**^2^**, Samuthira Nagarajan^1,*^**

^1^ Department of Chemistry, Central University of Tamil Nadu, Thiruvarur- 610 005, India.

^2^ Department of Chemistry, Islamiah College, Vaniyambadi - 635 752, India.

**Synthesis details**

**Synthesis of 1,2-Di-(1-naphthyl)ethane**^1^ **2**

1-(Chloromethyl)naphthalene **1** (14.2 g, 80 mmol) in dry THF (50 mL) was taken in a dropping funnel and added dropwise to the suspension of Mg turnings (1.07 g, 44 mmol) in dry THF (5 mL) at room temperature. The reaction mixture was stirred at room temperature for about 2 h and then refluxed for half an hour. Methanol was added to quench the reaction and the reaction mixture was filtered through suction. The filtrate was diluted with CHCl_3_, washed with water and dried in Na_2_SO_4_.The solvent was removed under reduced pressure and the residue was washed with methanol to afford dinaphthylethane **2** as white color solid (7.30 g, 60 %). ^1^H NMR (400 MHz, CDCl_3_): δ (ppm) 7.27-7.93 (m, 14 H),3.17 (s, 4H). HRMS (ESI): m/z calcd for C_22_H_18_ [M+H] 283.1481, found 283.2643.

**Synthesis of Picene ^2^ 3**

A solution of (2 g, 7 mmol) of dinaphthylethane **2** in 12 mL of carbon disulfide was boiled for two hours on the water bath with of anhydrous aluminium chloride (4 g, 30 mmol). The progress of the reaction was monitored by TLC. After the completion of reaction, the reaction mixture was poured into the ice cold water and then extracted with chloroform. The organic layer was dried over Na_2_SO_4_ and the solvent was removed under reduced pressure. The resulting solid was purified by column chromatography (silica, eluent: hexane) to afford picene **3** as an off-white solid (0.8 g, 40%).^1^H NMR (400 MHz, CDC1_3_): δ (ppm) 9.01 (s, 2H), 8.91 (s, 2H), 8.84 (m, 2H), 8.46 (m, 2H), 7.81 (m, 2H), 7.78 (m, 2H). HRMS (ESI): m/z calcd for C_22_H_14_ [M+H] 279.1096, found 279.1582.

**Synthesis of N,N′-di(dodecyl)-perylene3,4,9,10-tetracarboxylic diimide** ^3^ **6**

A mixture of 3,4,9,10-perylenetetracarboxylic acid anhydride **4** (0.800 g, 2.03 mmol), dodecylamine **5** (1.2 mL, 5.07 mmol), and imidazole (6 g) was stirred at 160 °C for 5 h. After cooling to room temperature, ethanol (30 mL), hydrochloric acid (20 mL) and water were added and then stirred overnight. The resulting solid was collected by filtration; the filter cake was washed with water until pH 7. The solid was dried, dissolved in a small amount of chloroform and passed through short pad of silica. C12-PTCDI **6** was obtained as dark brown solid^3^ (0.93 g, 95%). ^1^H NMR (400 MHz, CDCl_3_): δ (ppm) 8.70 (d, J=8Hz, 4H), 8.63(d, J= 5.6Hz, 4H), 4.23 (t, J=12.8 Hz, 4H), 1.80 (t, J=12.8Hz, 4H), 1.75-1.25 (m, 36H),0.906 (t, J=20 Hz, 6H). HRMS (ESI): m/z calcd for C_48_H_58_N_2_O_4_ [M^+^] 726.4397, found 726.4408.

**Optical properties**

**
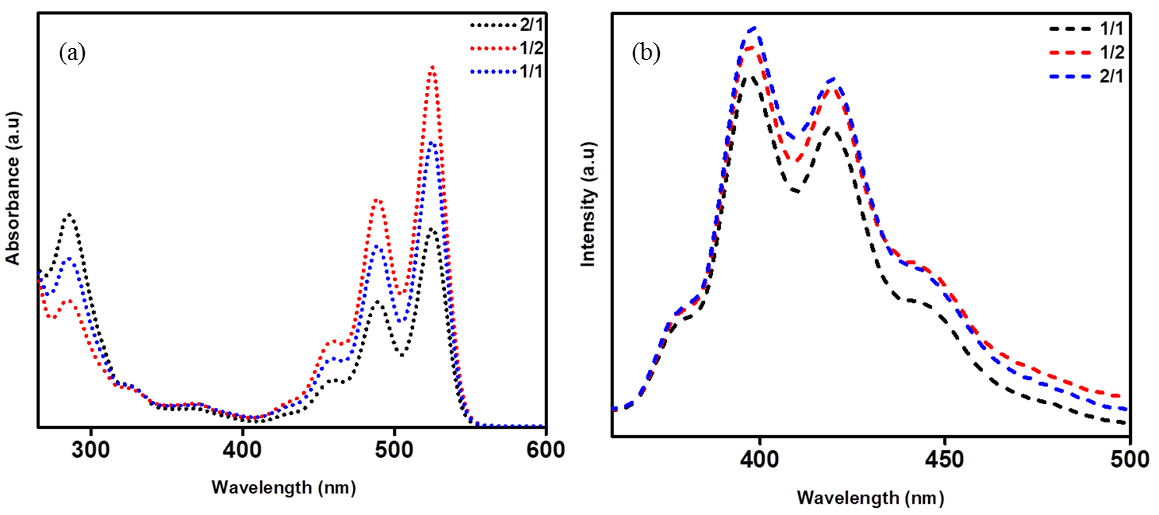
**

**Figure S1.**a) Absorption (10^-3^ M) b) emission spectra (10^-5^M) of D/A blends

**Computational Studies**

To evaluate the electronic properties of the pristine **D** and **A** molecules in ground state density functional theory (DFT) has been employed.^4, 5^ The calculations were performed in Gaussian (6-31 d) and Vienna ab initio simulation package (VASP) at local density approximation (LDA) and generalized gradient approximation (GGA) theory. To involve the solvent correction role, the PBE were performed (D2 scheme). The electronic and material properties were calculated from MedeA based on the optimized parameters. The optimized parameters include the crystalline constraints such as sides and angles. Based on these single crystal parameters, polycrystals were built for various symmetries available in Discovery Studio software. The polycrystals were predicted for a few popular space groups p21/c, p2, pbca, pna2, cc, etc. (Cambridge Structural database reference). All the space groups were tried sequentially and the interacting/packing measurements were monitored. Optimal lattice distances (such as interatomic distances and dipole moments) were obtained from right packing and finalized for study.

The DFT studies are extended to visualize the frontiers molecular orbitals (FMOs) of pristine donor and acceptor. The highest occupied molecular orbital (HOMO) and lowest unoccupied molecular orbital (LUMO) energy levels are given in Table S1. The distributions were obtained and shown in Figure S2. The HOMO and LUMO distribution is spread over the central part of the molecule and suggests easy charge carrier transport.


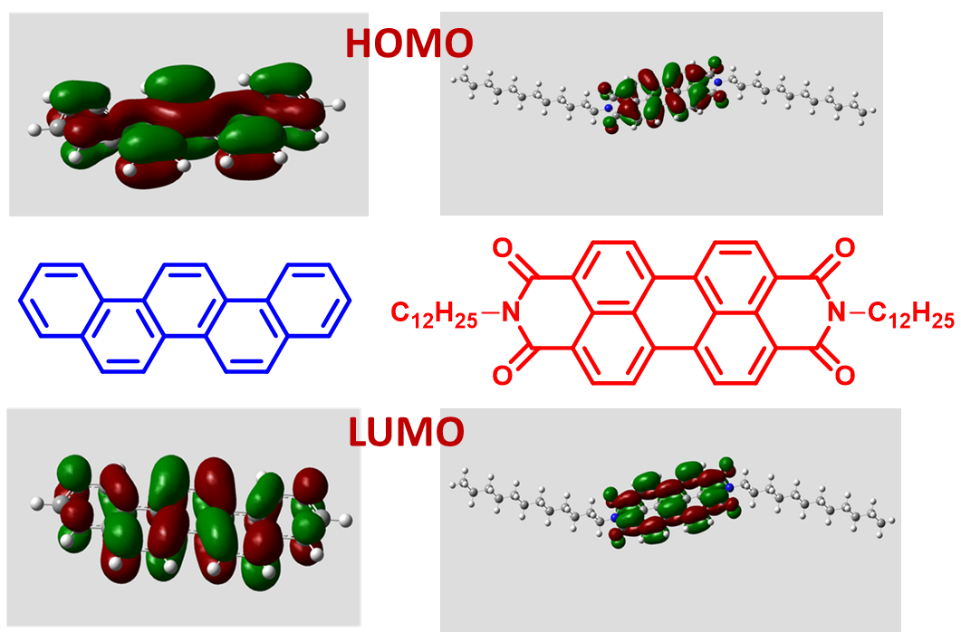


**Figure S2**. FMO of picene and C12-PTCDI

**Table S1**.HOMO, LUMO energy levels by DFT analysis.

| **Compounds** | **HOMO (eV)** | **LUMO (eV)** | **Band gap (eV)** |
| --- | --- | --- | --- |
| **Picene** | -5.53 | -1.25 | 4.288 |
| **C12-PTCDI** | -6.17 | -3.68 | 2.48 |

**Proposed molecular stacking**


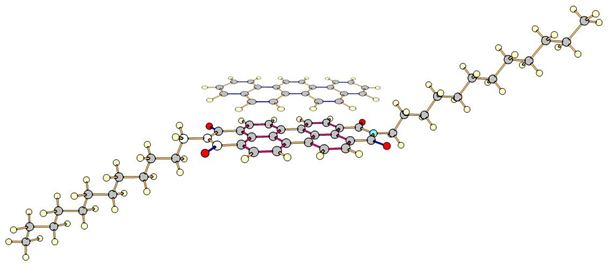
According to molecular mechanics calculation of the donor-acceptor molecule using MedeA's VASP, the packing of the D-A molecule was done.

**Figure S3**.The possible arrangement of D and A


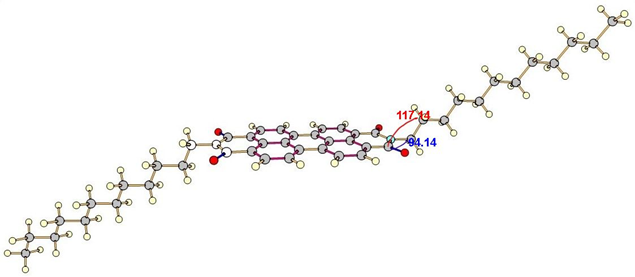


**Figure S4**. The dihedral angle of the imine nitrogen center

**(a)**

The electron mobility values were calculated from re-organisation energy based on optimization using Gaussian for Neutral and Cation /anion geometry and energy calculations of cation geometry and neutral geometry as per the formula

k = V^2^/ħ × sqrt(π/(λk_B_T)) × exp(-λ/(4k_B_T)) Where,

V=1/2 [E_homo_-E_homo-1_] for hole mobility and

V=1/2 [E_lumo+1_ – E_lumo_] for electron mobility

λ = (E_anion_(neutral geometry) - E(neutral)) + (E_neutral_(anion geometry) - E(anion))

Likewise, the hole mobility was calculated using the cation geometry and tabulated. (Table S2).

| **Table S2.** Hole and electron mobilities(theoretical) | | |
| --- | --- | --- |
| **Parameters** | **Picene** | **C12-PTCDI** |
| **Hole mobility** | 0.3806 cm^2^/v/s | - |
| **Electron mobility** | - | 0.1149 cm^2^/v/s |
| **V** | 0.1492 | 0.7782 |
| **Λ** | 0.1908 eV | 3.0751 eV |

The other part of the calculation using VASP gave us insight into the crystalline pattern of the molecules. The Fermi bands and density of states (DOS) provided information about possible space charge carrier can move. DOS are represented as graphs as shown in Figure S5. The broadening of peaks can be attributed to hybridization. When the orbitals of the donor are doped with the acceptor, the layers are further hybridized and provide more bands for transportation of the charge carrier with less energy.

**
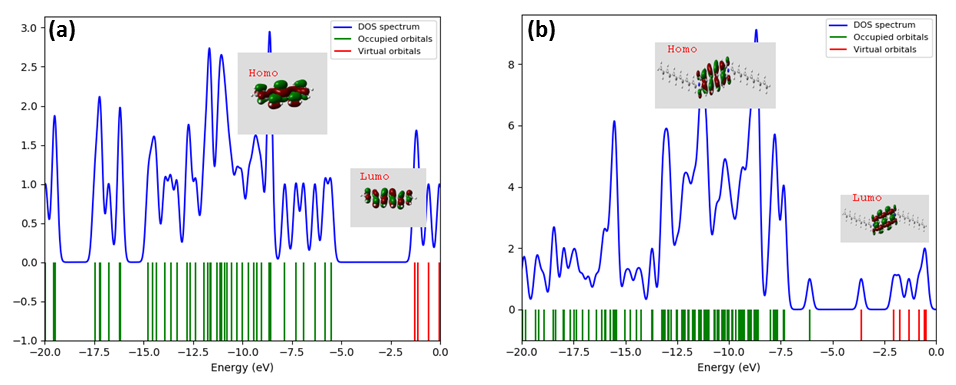
**

**Figure S5.** Density of states for (a) donor and (b) acceptor

The **D-A** complex DOS is shown in Figure S6 explains the complexity in energy levels. Here the axes have been reversed for a clear Fermi gap representation. The light blue and deep blue regions indicate the levels near the core and near the band gap respectively, as a result of mixing. The black peaks represent the enhanced Fermi levels as a result of complexing. In the y-axis, the virtual levels were occupied by mixed orbitals extending up to a value of 150 (1/eV: x-axis)


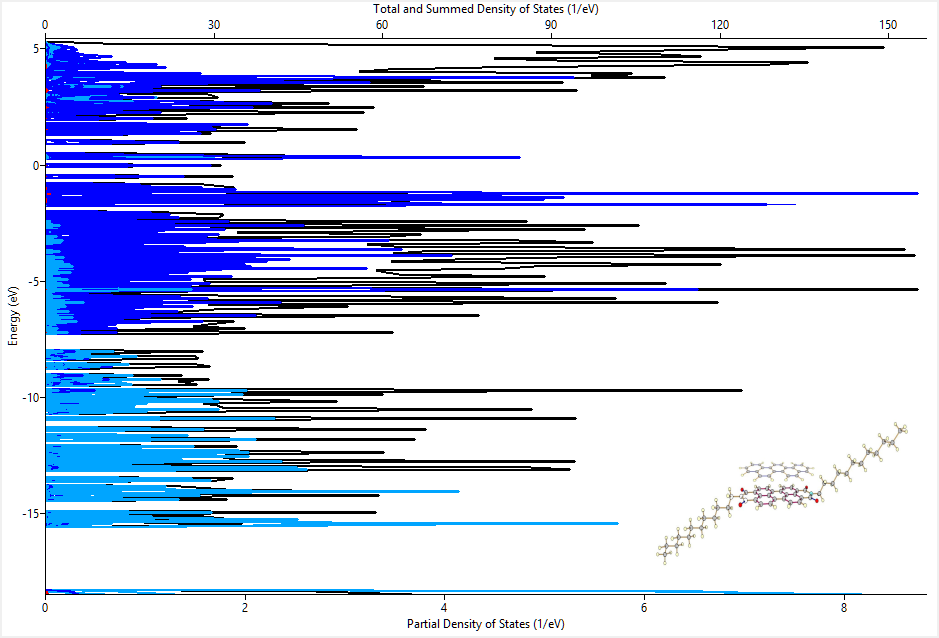


**Figure S6**. Density of states distribution for **D:A** in 1:1 ratio

**Time dependent DFT studies**

Further, from time dependent DFT studies the spectral behaviour of donor and acceptor molecules have been investigated. Especially, donor molecule has more number of accessible orbitals which are located over the entire ring. A close agreement was observed for the band gap and the excited state energy of **D** and **A**. These values are given in **Table S3**. Band gap value of 4.28 eV is pertaining to donor’s transition at 289 nm, corresponding to HOMO-2 to LUMO+2 in a triplet state. The other possible energy levels for donor are ranging from 3.4 to 4.6 eV.

| **Table S3**. Possible electronic transitions for D and A predicted by TD-DFT. | | | | |
| --- | --- | --- | --- | --- |
| **D/A** | **Absorptionλ_abs_(nm)** | **Electronic Transition** | **Energy (eV)** | **Wavelength (nm)** |
| **A** | 525 | S_0_-S_1_ | 2.427 | 510.86 |
| **A** | 486 | S_0_-T_1_ | 2.643 | 468.98 |
| **A** | 456 | S_0_-T_3_ | 2.777 | 446.34 |
| **D** | 265-325 | S_-1_-T_2_  S_0_-S_1_  S_1_-T_4_  S_-4_-T_1_ | 3.401  3.835  3.854  4.659 | 364.49  323.25  321.63  266.09 |
| **D** | 289 | S_-2_-T_2_ | 4.287 | 289.15 |


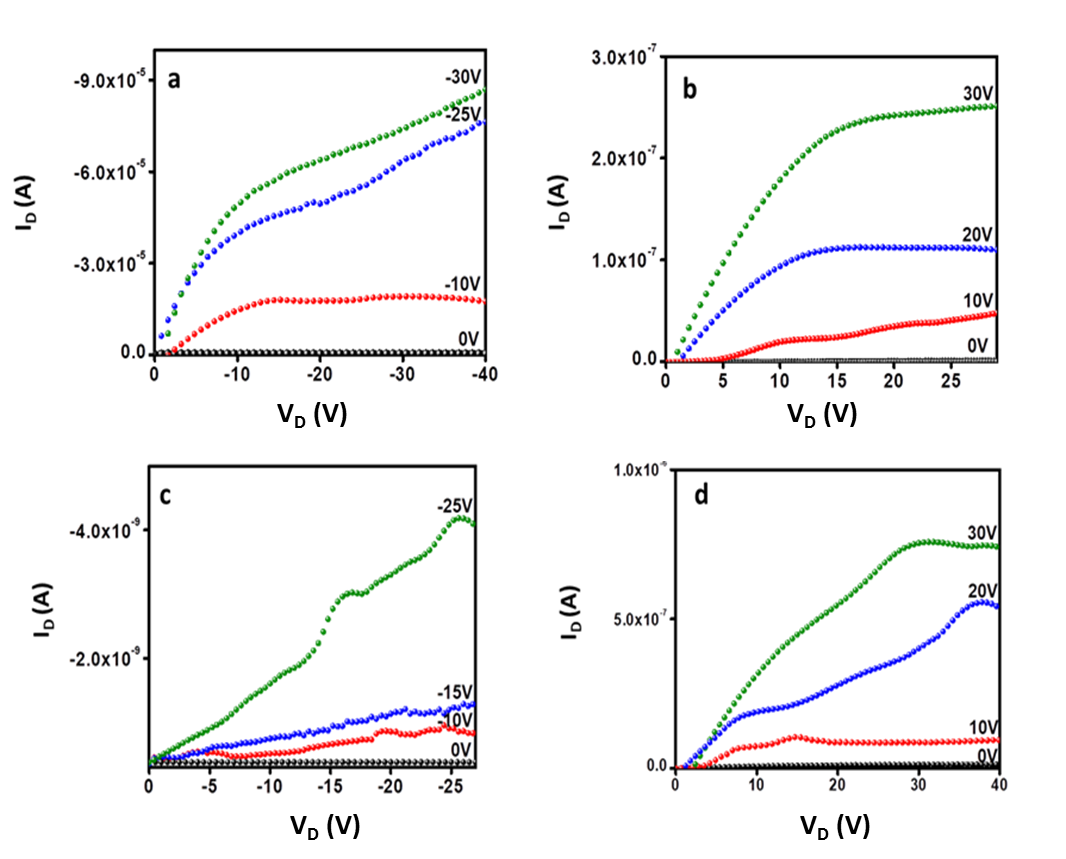

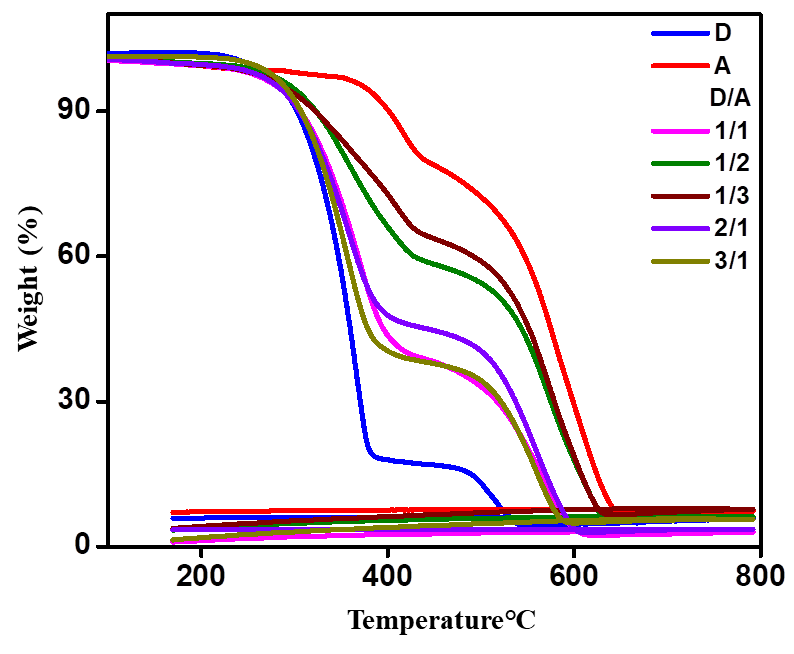


**Figure S8**. Output characteristics of 1/2 (a,b) &1/3(c,d) blends

**Figure S7**. TGA curves of **D**, **A** and **D/A** blends


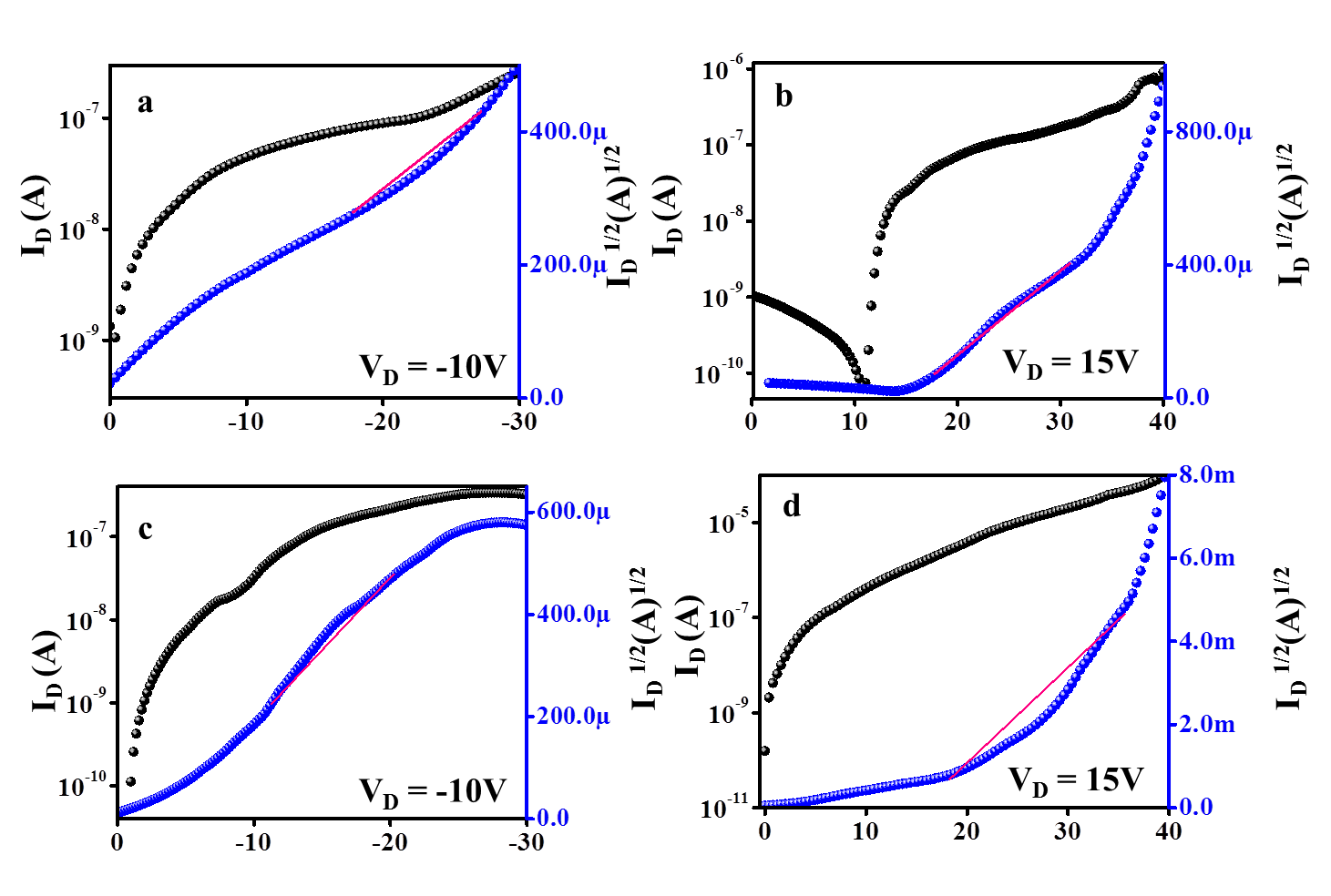
**
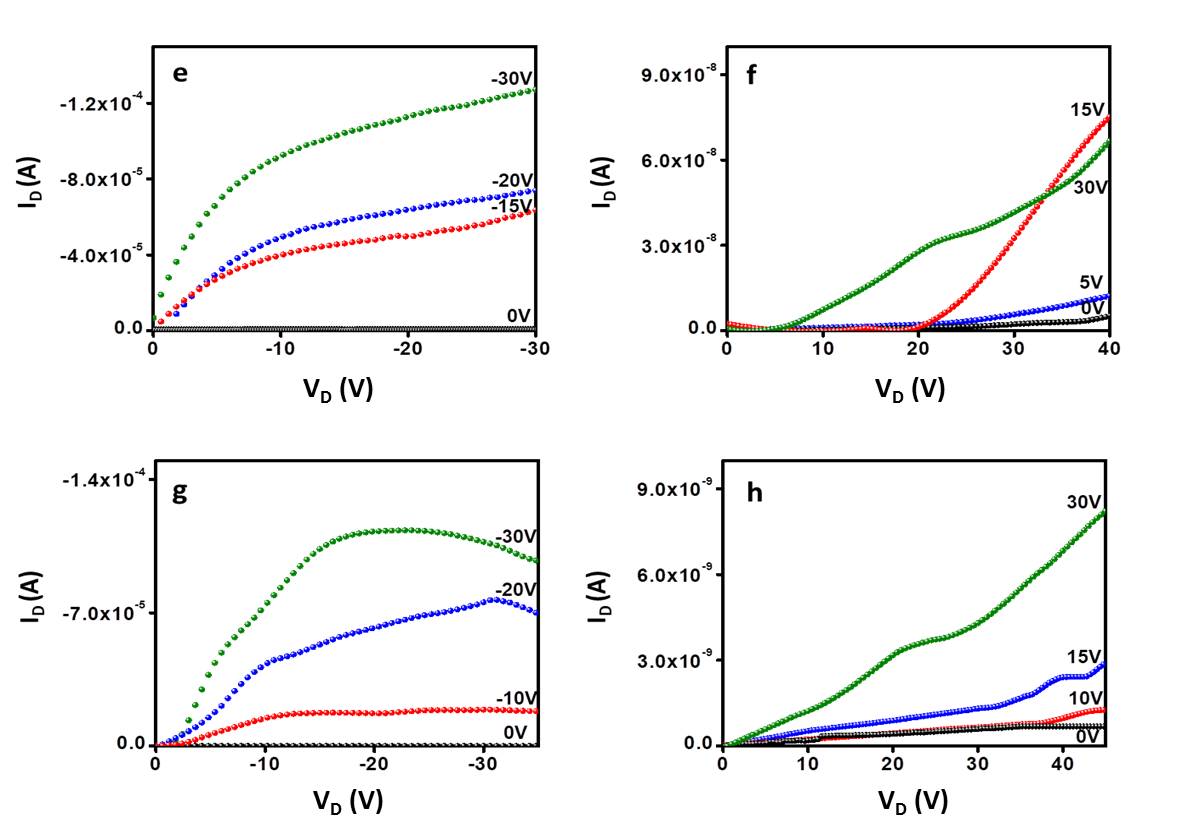
**

**Figure S9**. Output characteristics of 2/1 (c,d) & 3/1(e,f) blends

**Figure S10**. Transfer characteristics of 1/2 (a, b) & 1/3 (c, d) blends

**
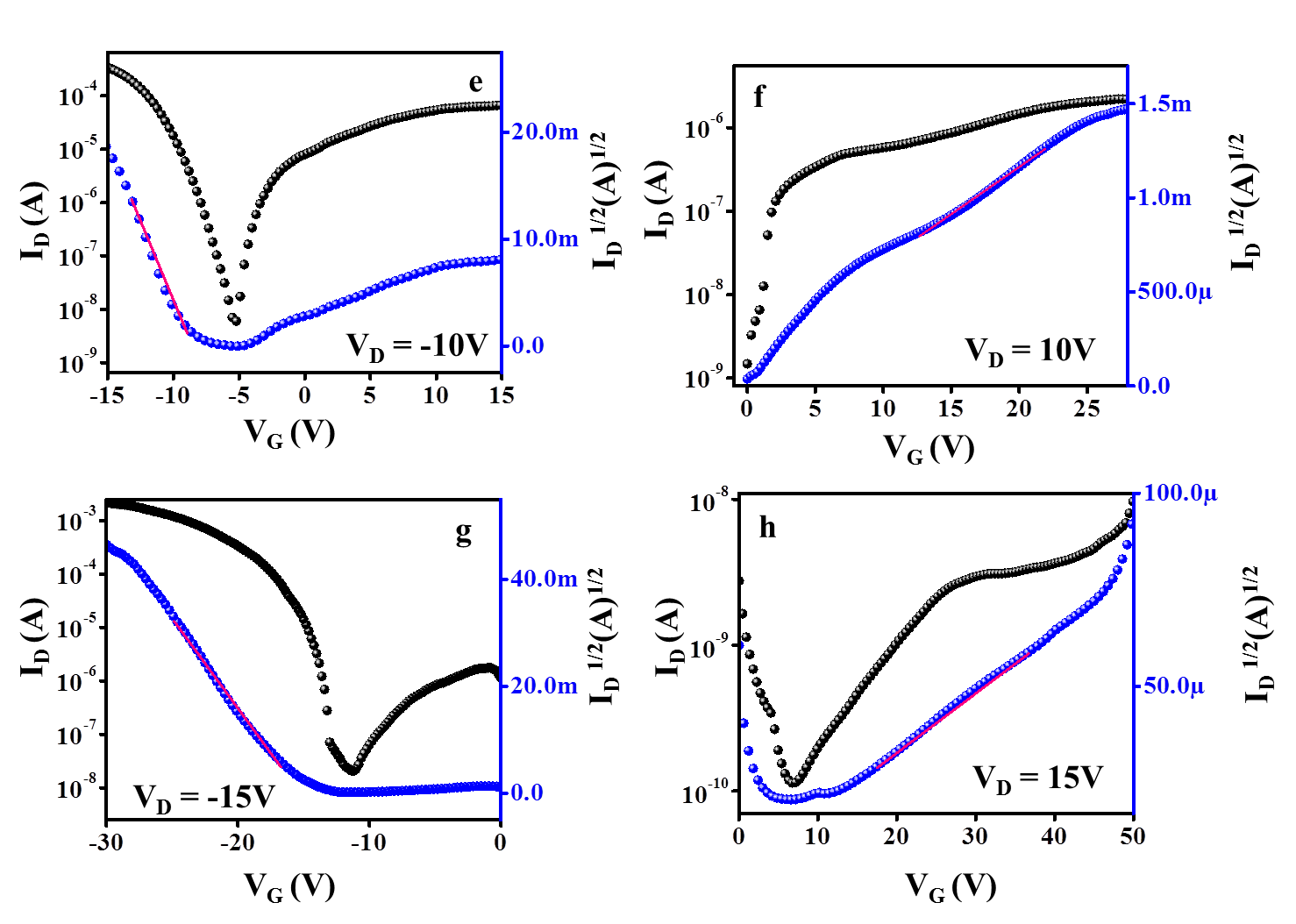
**

**Figure S11**. Transfer characteristics of 2/1 (e, f) & 3/1 (g, h) blends

**2**

**Figure S12**. ^1^H NMR spectrum of compound **2**


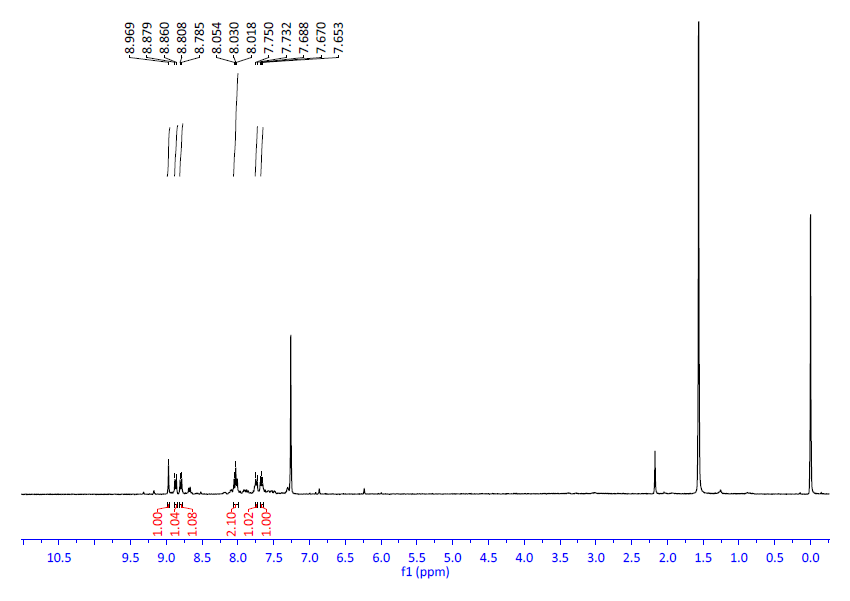

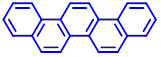


**Figure S13**. ^1^H NMR spectrum of compound **3**


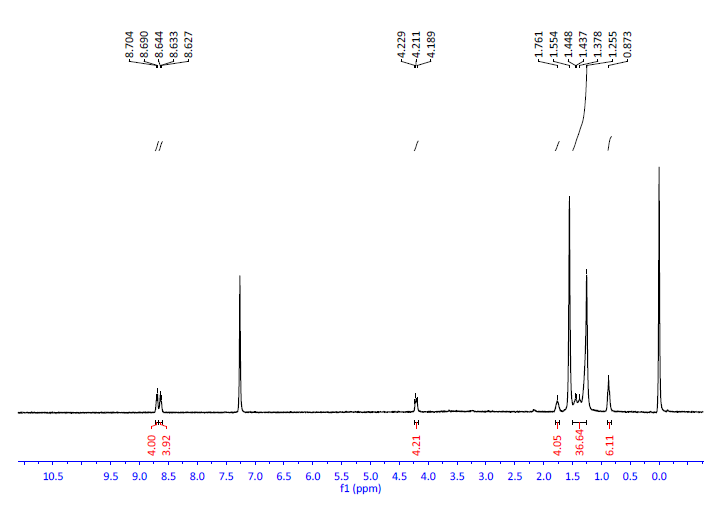

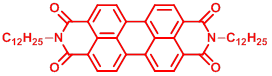


**6**

**3**

**Figure S14**. ^1^H NMR spectrum of compound **6**


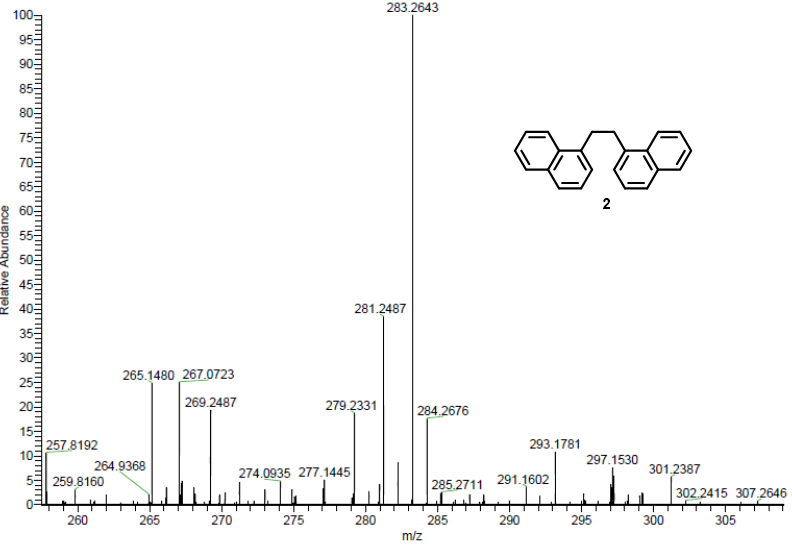


C_22_H_18_ [M+H] calcd283.1481, found 283.2643

**Figure S15**. HR- mass spectrumof compound **2**

**Figure S15**. HR- mass spectrum of compound **2**

**Figure S16**. HRMS spectrum of compound **3**


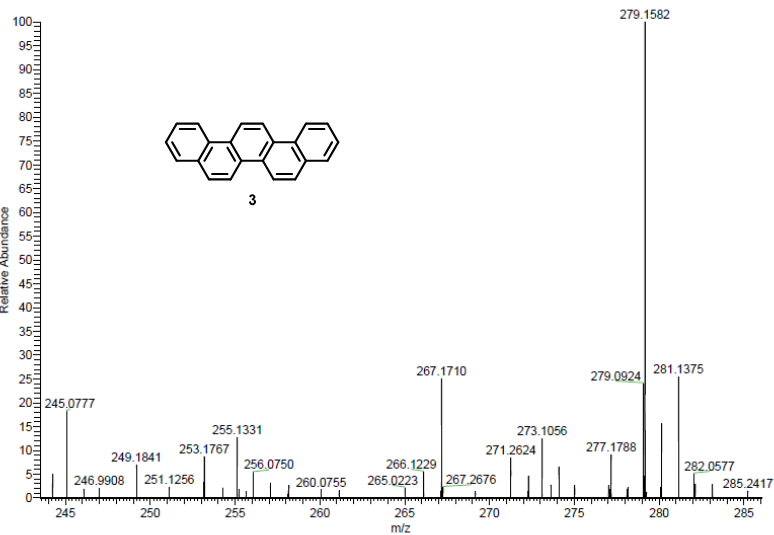


C_22_H_14_ [M+H] calcd 279.1096, found 279.1582

**Figure S16**. HR- mass spectrum of compound **3**


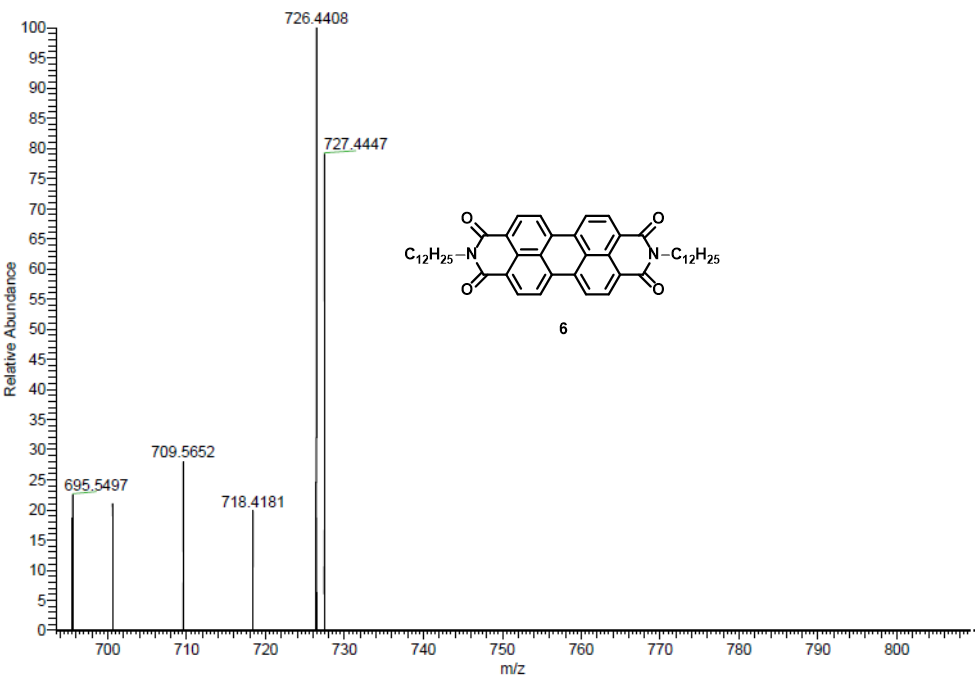


C_48_H_58_N_2_O_4_ [M^+^] calcd726.4397, found 726.4408

**Figure S17**. HR- mass spectrum of compound **6**

References:

(1) Okamoto, H.; Yamaji, M.; Gohda, S.; Kubozono, Y.; Komura, N.; Sato, K.; Sugino, H.; Satake, K. Facile Synthesis of Picene from 1,2-Di(1-naphthyl)ethane by 9-Fluorenone-Sensitized Photolysis.Org. Lett. **2011**, *13*, 2758.

(2) Buu-Hoi, N. P.; Hoan, N. The Reaction of α-Halogenated Arylalkanes with Metal Powders in Hydroxylated MediaJ. Org. Chem. **1949**, *14*, 1023.

(3) Boobalan, G.; Imran, P.K.M.; Nagarajan, S. Self-assembly and Optical Properties of *N* *N*′-bis(4-(1-benzylpiperidine))perylene-3,4,9,10-tetracarboxylic diimide. Supramol. Chem. **2012**, *24*, 238.

(4) J. Harl and G. Kresse. Accurate Bulk Properties from Approximate Many-Body Techniques. Phys. Rev. Lett. **2009**, *103*, 056401.

(5) Zhu, F.; Zhang, Q.; Zhou, J.; Li, H.; Lu, J. Tuning the Microstructure of Donor/Acceptor Blend Films To Achieve High-Performance Ternary Data-Storage Devices. J. Phys. Chem. C. **2019**, *123*, 12154–12160.
